# Chironomidae (Diptera) of the Šventoji and Žeimena sub-basins in Lithuania

**DOI:** 10.3897/BDJ.12.e130218

**Published:** 2024-11-08

**Authors:** Laurynas Stasiukynas, Fabio Laurindo da Silva, Jekaterina Havelka, Sigitas Podėnas, Aistė Lekoveckaitė

**Affiliations:** 1 Vilnius University, Life Sciences Center, Vilnius, Lithuania Vilnius University, Life Sciences Center Vilnius Lithuania; 2 Universidade de São Paulo, São Paulo, Brazil Universidade de São Paulo São Paulo Brazil

**Keywords:** Chironominae, Diamesinae, Orthocladiinae, Prodiamesinae, Tanypodinae, checklist, streams

## Abstract

**Background:**

Chironomidae, commonly known as non-biting midges, are key indicators of the health and biodiversity of freshwater ecosystems. They are also one of the most abundant and diverse groups of aquatic invertebrates. Although Chironomidae are ecologically important, abundant and diverse, there has been limited focused research on this group in Lithuania. Our study addresses this gap by analysing the diversity of Chironomidae in six Lithuanian streams.

**New information:**

In this study, we present a comprehensive list of Chironomidae collected from six streams with similar hydrological characteristics: three dammed and three undammed. Lithuania is home to 158 species of known species of Chironomidae, comprised of 22 species in the subfamily Tanypodinae, 87 in the Chironominae, 42 in the Orthocladiinae, four in the Diamesinae and three in the Prodiamesinae. Throughout our research, we collected 11,296 chironomid specimens using a D-shaped aquatic net. Amongst these specimens, we identified 89 species representing 65 genera and five subfamilies, including 37 species and 12 genera, were recorded for the first time in Lithuania. The subfamily Chironominae, with 28 genera and 6,816 specimens, has exhibited the highest abundance of non-biting midges both in terms of genera and individuals. Amongst the streams investigated, the Luknelė was the richest in diversity, harbouring 37 genera and 2,657 individuals, accounting for about 55% of the Chironomidae genera found during the research. Our findings significantly enhance the understanding of the Lithuanian Chironomidae fauna, marking the first comprehensive study of such a kind, as previous knowledge of this fauna has been derived only from related studies.

## Introduction

Rivers and streams occupy only 0.51% of Lithuania's territory (Gailiušis et al. 2001). The country boasts approximately 29,000 streams longer than 0.25 km, with a cumulative length of about 64,000 km (Kilkus and Stonevičius 2011). Around 80% of these streams (representing about 51% of the total stream length) are very small, measuring up to 3 km in length (Jablonskis et al. 2007). There are 3,646 small streams ranging from 3 to 10 km, collectively accounting for 24.5% of the total stream length. Medium-sized rivers, ranging from 10 to 100 km, constitute 20.4% of the total stream length, with a total of 75 such streams present in the country. Notably, only 17 rivers in Lithuania exceed 100 km in length, comprising just 0.1% of the total stream length. The Nemunas Basin is the largest river basin in Lithuania, covering an area of 46,695.4 km² within the country, with a total basin area of 97,863.5 km². The Nemunas River, flowing through this Basin, is the longest river in Lithuania, stretching for 475 km within Lithuania out of its total length of 937.4 km. The investigated streams are part of two sub-basins. The first sub-basin is the Šventoji River, the longest river flowing entirely within Lithuania, with a length of 246 km and a basin area of 6,889 km^2^. The second sub-basin is the Žeimena, with Žeimena River measuring 79.6 km in length and encompassing a basin area of 2,793 km^2^ (Jablonskis et al. 2007).

Diptera is the predominant group of macroinvertebrates in freshwater ecosystems, typically comprising the largest biomass and it is also one of the most extensively researched groups in freshwater environments (Ivković et al. 2020). Amongst aquatic macroinvertebrates, the Chironomidae family is generally the most abundant, both in individual numbers and species diversity (Farias et al. 2012, Rocha et al. 2012, Dantas et al. 2024). With over 6,000 species inhabiting various biotopes and occupying diverse niches, non-biting midges exhibit a wide array of trophic specialisations and lifestyles (Stur and Ekrem 2020). Given their tendency to dominate freshwater fauna, Chironomidae are considered important bioindicators for assessing freshwater (Lencioni et al. 2012). Consequently, they play a crucial role in monitoring, protecting and conserving freshwater environments (Cortelezzi et al. 2020).

In Lithuania, more than 150 species of Chironomidae have been recorded (Pakalniškis et al. 2006, Ruginis 2007, Móra and Kovács 2009). However, only a few isolated identifications have been made in previous studies (Móra and Kovács 2009) and comprehensive research on this family is yet to be conducted in Lithuania. Based on studies of Chironomidae diversity in the neighbouring countries, it is likely that several times more species exist in the Lithuania (Schartau et al. 2010, Paasivirta 2014). The aim of this study was to expand knowledge about the diversity and distribution of Chironomidae in streams of Lithuania.

## Materials and methods

**Hydrography and geography of the sampling area**

**Figure 1.**
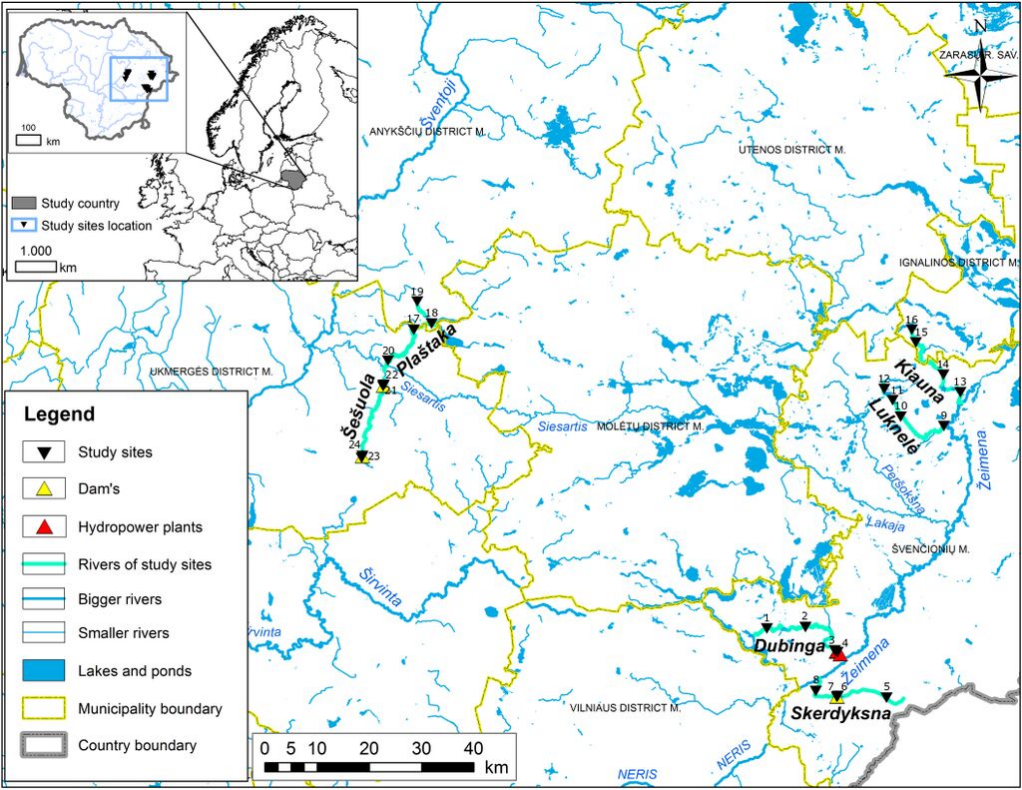
Map of Lithuania highlighting the studied streams and their locations.

The study was conducted in Lithuania, specifically within the Nemunas Basin, focusing on the sub-basins of the Šventoji and Žeimena Rivers. To ensure accurate data for the comparison, over 200 rivers and streams in Lithuania were screened and evaluated, based on data from the Rivers, Lakes and Ponds Cadastre of the Republic of Lithuania (UETK) data (Lietuvos Respublikos aplinkos ministerija 2024). Chironomidae specimens were collected from two types of streams: dammed (Skerdyksna, Šešuola, Dubinga) and undammed (Plaštaka, Kiauna, Luknelė). The streams were grouped into three pairs and then selected, based on their similarities in terms of geographic location, morphometric and hydrographic characteristics. The sample collection sites are situated in three protected areas: Anyksčiai and Asveja Regional Parks (Plaštaka, Šešuola, Dubinga and Skerdyksna) and Aukštaitija National Park and Labanoras Regional Park (Luknelė and Kiauna) (Fig. 1). All the streams are in the central-eastern part of Lithuania, with altitude ranges from 80 m to 150 m above sea level and traverse various landscapes, including natural or semi-natural forests and meadows, as well as agricultural and urbanised areas. The flow rate varied between the study sites, with a steady increase downstream in the undammed streams. However, the dammed streams varied considerably. For example, the Skerdyksna stream in the upper reaches passes through agricultural fields and is reclaimed, resulting in very low flow velocities. In the middle of the stream, at study sites 6 and 7, the stream is dammed. Upstream of the dam, a pond is formed, leading to stagnant water flow. Downstream of the dam, the flow velocity increases and the stream follows a natural course, characterised by a habitat-rich environment. The Šešuola stream is dammed twice, so all study sites were chosen at the dams. Study sites 23 and 21 are located in the ponds, resulting in stagnant water flow. Study site 24 is situated below the dam and in agricultural fields, where the stream section is reclaimed, with consistently low water levels averaging about 15 cm, leading to very low water velocity. Below the second dam, at study site 22, the river section is natural, which increases the flow rate. However, due to the dam and the varying rainfall, the stream depth fluctuates significantly during the season, ranging from 5 cm to 30 cm. The Dubinga stream, the third dammed stream, is dammed only in its downstream, while the upstream is natural or semi-natural, flowing through several small villages without intensive agriculture. The stream is quite deep in the upper and middle reaches, with study sites 1 and 2 maintaining an average depth of around 40 cm throughout the season. The last two survey sites in this stream were at the dam, with survey site 3 located in a pond, resulting in a stagnant water flow. Behind the dam, the stream's velocity and depth varied due to dam operations, with the depth ranging from 0 cm to 15 cm, causing highly variable flow velocity. The substrate of the studied streams varied not only between different streams, but also within the same stream at different study sites. Sand was the predominant substrate overall, but the composition and distribution of substrates varied between habitats. The substrate composition at the study sites included: silt, clay, mud, sand (the predominant substrate), granules, pebbles, cobbles and boulders. Additionally, some of the pristine sites were rich in detritus, with layers up to 0.5 m thick and contained dead wood trunks, some banks also being covered with abundant aquatic vegetation (Table 1).

**Sampling and identification**

**Figure 2.**
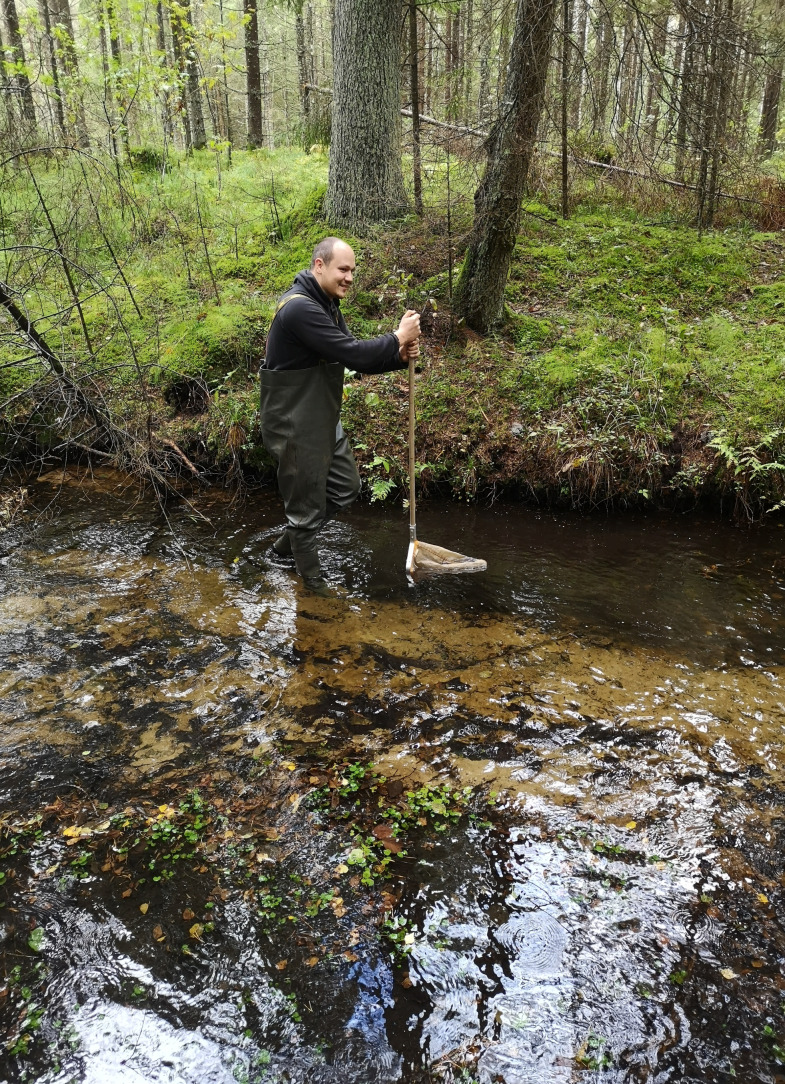
Sample collection using the Kick Sampling method by using D-shaped aquatic net.

The research was conducted in 2021 and 2022, spanning from May to September. Sampling was conducted across six streams with four research sites in each, resulting in sampling from 24 sites in total. Samples were gathered every two weeks using a D-shaped aquatic net mesh size of 1 mm (Fig. 2). At each site, a 1 m² area was randomly selected for sampling, which was conducted using the Kick Sampling method (Letovsky et al. 2012). Samples collected were transferred into 2 litre zip-lock bags filled with 99% propylene glycol. Upon collection, all samples were stored in a refrigerator at 4°C in the Life Sciences Centre of Vilnius University. Subsequently specimens which were identified as belonging to the Chironomidae family underwent meticulous separation and were preserved in containers filled with 97% ethanol.

Chironomidae larvae were identified by using taxonomic keys, based on morphology (morpho) available from Epler (2001), Orendt et al. (2011) and Andersen et al. 2013. The systematics and nomenclature of taxa follow Andersen et al. (2013). For molecular analysis, larvae from 200 specimens were selected. Total genomic DNA was extracted from each larva using the DNeasy Blood and Tissue kit (Qiagen) according to the manufacturer's protocol. Partial sequences of the mitochondrial cytochrome c oxidase subunit I (COI) gene were amplified using primers LCO-1490 and HCO-2198 (Folmer et al. 1994). PCR amplification was performed in a thermal cycler (Eppendorf) in 30 μl reaction volumes containing 3 μl genomic DNA, 1.5 μl of each primer (0.5 μM), 15 μl of DreamTaq PCR Master Mix (Thermo Scientific) and 9 μl of nuclease free water (Thermo Scientific). The cycling parameters were as follows: initial denaturation at 95°C for 3 minutes (1 cycle), denaturising at 95°C for 30 seconds, annealing at 49°C for 30 seconds, extension at 72°C for 60 seconds (35 cycles in total) and final extension at 72°C for 10 minutes (1 cycle). PCR products were purified using the GeneJet PCR purification kit (Thermo Scientific) and sequenced at Macrogen Europe BV (Amsterdam, the Netherlands). The amplification primers were also used as sequencing primers. DNA sequences for each specimen were aligned in the BioEdit Sequence Alignment Editor (Hall 1999) and compared by BLAST (National Library of Medicine 2024). GenBank accession numbers for each individual are given in the Check List.

Sample-based rarefaction curves were produced to visually compare the genera richness of non-biting midges amongst the researched streams and to assess the sampling effort (Gotelli and Colwell 2001). The similarity of chironomid assemblages collected over two study years from dammed and undammed streams was visualised using non-metric multidimensional scaling (NMDS) ordination, based on the Bray–Curtis similarity index. The analyses were conducted using PAST 4.07b software (Hammer et al. 2001).

## Checklists

### Check list of Chironomidae collected across six streams: Skerdyksna, Šešuola, Dubinga, Plaštaka, Kiauna and Luknelė

#### Diptera

Linnaeus, 1758

A87EC1B0-147E-5803-8488-8CAD317598D3

#### Nematocera

Dumeril, 1805

40D8C977-4B6C-590D-BC6C-C6D0EABE4EE4

#### Culicomorpha

Hennig, 1948

EBF0AD26-7139-5971-A2BA-65123DD84A68

#### CHIRONOMIDAE

Newman, 1834

E8CB2337-19F7-55C4-B893-B710E52F9944

#### Tanypodinae

Skuse, 1889

E699DE60-B3F5-5E82-8C69-19E6F1D0CC91

#### Anatopyniini

Fittkau, 1962

6A743EB0-3533-5779-A6D4-7EB80A244D1A

#### Anatopynia

Johannsen, 1905

4D511B14-057E-50D6-9249-BA75A451F569

#### Anatopynia sp.

77109D43-AD32-5C79-8A47-073DA429DBE8

##### Notes

Morpho identification. First record for Lithuania (Lapinskaitė 1968).

#### Coelotanypodini

Coffman, 1978

21F91EE4-6A4D-58AC-88B8-3428AF90FA3D

#### Clinotanypus

Kieffer, 1913

38F143E9-D344-5A10-86E3-93CBCA643459

#### Clinotanypus nervosus

(Meigen, 1818)

0C5E725A-2CD2-51CC-BAF2-02FE157FA990

##### Notes

Morpho-molecular identification (GenBank ID: PQ458089; PQ458090; PQ458091). First record for Lithuania (Lapinskaitė 1968).

#### Macropelopiini

Zavřel, 1929

07DA0A2C-F0BF-5DE5-9A71-DB6381B40EBF

#### Apsectrotanypus

Fittkau, 1962

8762F45F-578F-589B-ABCA-05C7EC3D23D7

#### Apsectrotanypus trifascipennis

(Zetterstedt, 1838)

31FDDD94-AF5B-5BB8-B1A2-DFA317120F72

##### Notes

Morpho-molecular identification (GenBank ID: PQ458073; PQ458074). First record for Lithuania (Móra and Kovács 2009).

#### Macropelopia

Thienemann, 1916

22EBE0B5-EB00-55B4-9753-A3880395C650

#### Macropelopia nebulosa

(Meigen, 1804)

D0A46518-8725-52C4-A9B0-4035D0D4C1DF

##### Notes

Morpho identification. New record for Lithuania.

#### Macropelopia notata

(Meigen, 1818)

DA8161E7-7CFD-5D24-A293-9CF27E2BB06D

##### Notes

Morpho identification. First record for Lithuania (Sæther and Spies 2005).

#### Psectrotanypus

Kieffer, 1909

E607F270-3FDF-5952-B40A-A6B7F18B25FD

#### Psectrotanypus varius

(Fabricius, 1787)

3E83C783-ED47-5A8D-B0CB-F2EDB7B59C4F

##### Notes

Morpho-molecular identification (GenBank ID: PQ458163). First record for Lithuania (Grigelis 1999).

#### Pentaneurini

Hennig, 1950

B75599DC-5481-59C3-A8B7-ED0663591D0D

#### Ablabesmyia

Johannsen, 1905

8CAC0A0B-32CC-5DF7-866B-C87C9B8AD21E

#### Ablabesmyia (Ablabesmyia) longistyla

Fittkau, 1962

301E6A45-F32E-5A52-A547-BA91B4879B51

##### Notes

Morpho-molecular identification (GenBank ID: PQ458066; PQ458067; PQ458068; PQ458069; PQ458070; PQ458071). First record for Lithuania (Sæther and Spies 2005).

#### Ablabesmyia (Ablabesmyia) monilis

(Linnaeus, 1758)

38CAF0D2-E115-5B15-9365-D9B251A49065

##### Notes

Morpho identification. First record for Lithuania (Grigelis 1999).

#### Ablabesmyia (Ablabesmyia) phatta

(Egger, 1864)

7B46420E-24C1-56DB-8777-3AA3124157BF

##### Notes

Morpho identification. First record for Lithuania (Grigelis 1999).

#### Conchapelopia

Fittkau, 1957

79A94CF8-3BD1-5867-A426-5EEBCB81281E

#### Conchapelopia melanops

(Meigen, 1818)

25C9B615-8901-535C-9AFD-ED08808514E7

##### Notes

Morpho-molecular identification (GenBank ID: PQ458092; PQ458093; PQ458094; PQ458095; PQ458096; PQ458097). New record for Lithuania.

#### Krenopelopia

Fittkau, 1962

F0BDB53E-892C-5C20-9ED7-F280BB74B07E

#### Krenopelopia binotata

(Wiedemann, 1817)

5AA4CC02-5132-596D-B2A4-03524821C8A2

##### Notes

Morpho identification. First record for Lithuania (Sæther and Spies 2005).

#### Larsia

Fittkau, 1962

050B00B2-57ED-59D2-9BAE-511BCFD7420F

#### Larsia atrocincta

(Goetghebuer, 1942)

8A8BBE6B-C6AB-51F7-8F99-CCA3F3BE45ED

##### Notes

Morpho-molecular identification (GenBank ID: PQ458114; PQ458115). New record for Lithuania.

#### Zavrelimyia

Fittkau, 1962

BB9A5F46-DE50-5DC0-9D1D-03E08C495639

#### Zavrelimyia melanura

(Meigen, 1804)

08B26561-EA5B-51B6-9949-CF7B09BA38AE

##### Notes

Morpho identification. First record for Lithuania (Pliūraitė and Kesminas 2004).

#### Procladiini

Roback, 1971

FE12EE47-84EB-50A3-9670-A82B681E5CEC

#### Procladius

Skuse, 1889

2FA59F78-134C-5FBE-8F94-C3E828F319DC

#### Procladius (Holotanypus) crassinervis

(Zetterstedt, 1838)

F4A59253-6B5C-5A14-A62D-8CB2BDCD3C07

##### Notes

Morpho-molecular identification (GenBank ID: PQ458151). New record for Lithuania.

#### Procladius (Holotanypus) culiciformis

(Linnaeus, 1767)

5D48AFE7-F6B3-52D5-A032-6A71EC47BBDA

##### Notes

Morpho-molecular identification (GenBank ID: PQ458152; PQ458153; PQ458154). New record for Lithuania.

#### Procladius (Holotanypus) denticulatus

Sublette, 1964

63FA4A05-815C-56BF-AF25-CF297CEE6E20

##### Notes

Morpho-molecular identification (GenBank ID: PQ458155; PQ458156; PQ458157). New record for Lithuania.

#### Procladius (Holotanypus) fuscus

Brundin, 1956

4D81C87E-E088-5A23-BAF3-5894D48E0854

##### Notes

Morpho identification. New record for Lithuania.

#### Procladius (Holotanypus) pectinatus

(Kieffer, 1909)

AA13A397-3AC1-53D4-AC3D-3E79145826E5

##### Notes

Morpho-molecular identification (GenBank ID: PQ458158). New record for Lithuania.

#### Procladius sp.

Skuse, 1889

F906BCEE-8782-502D-9EAC-EAF280B12452

##### Notes

Morpho-molecular identification (GenBank ID: PQ458159). First record for Lithuania (Grigelis 1999).

#### Tanypodini

Skuse, 1889

23D72B43-961E-5B81-9962-6C59A59BC09D

#### Tanypus

Meigen, 1803

452E4A59-F22D-5D81-AFD7-6CBE6AF034C6

#### Tanypus (Tanypus) kraatzi

(Kieffer, 1912)

8BA6687E-E4D1-57B7-AF42-A27F6E460E92

##### Notes

Morpho identification. First record for Lithuania (Grigelis 1999).

#### Tanypus (Tanypus) vilipennis

(Kieffer, 1918)

AA645735-90DD-57AE-9A83-3D0656859523

##### Notes

Morpho identification. First record for Lithuania (Grigelis 1999).

#### Diamesinae

Kieffer, 1922

C15D0E9C-DD1C-529F-AAE9-C5E866A31369

#### Diamesini

Kieffer, 1922

9A2A9D62-3D2C-510C-BE52-0F09F773168B

#### Potthastia

Kieffer, 1922

B6FF263F-C6D7-5A1B-B671-A3B51F9582BB

#### Potthastia sp.

1F45E13E-C598-5AAA-9C1C-A9FF98E998CC

##### Notes

Morpho identification. First record for Lithuania (Gasiūnas 1959).

#### Pseudodiamesa

Goetghebuer, 1939

CCDAB988-147D-5720-BEE9-F64584C2A3F0

#### Pseudodiamesa (Pachydiamesa) arctica

(Malloch, 1919)

7DBAC0B5-1AAA-5051-9DA2-0FC460BFF9AE

##### Notes

Morpho identification. First record for Lithuania (Grigelis 1999).

#### Prodiamesinae

Sæther, 1976

53156AD5-6F1F-5145-84C5-B9913BD9D958

#### Monodiamesa

Kieffer, 1922

F625A0C5-2DC9-5837-93E0-4FD7AC563FEE

#### Monodiamesa bathyphila

(Kieffer, 1918)

EDFA9DC5-DE59-53B5-BCAF-2C3E1E0B9F95

##### Notes

Morpho-molecular identification (GenBank ID: PQ458130; PQ458131). First record for Lithuania (Grigelis 1999).

#### Odontomesa

Pagast, 1947

BE8C717A-BF66-5DD0-9D6C-D0B53ADA0813

#### Odontomesa fulva

(Kieffer, 1919)

BD40D937-78D6-56C0-8C2E-C787EA3035C4

##### Notes

Morpho-molecular identification (GenBank ID: PQ458134). First record for Lithuania (Pliūraitė 2001b).

#### Prodiamesa

Kieffer, 1906

342CC376-E834-5D28-8B4B-C0AD217627E9

#### Prodiamesa olivacea

(Meigen, 1818)

A001A77F-5E38-5D70-9165-A680ACEB3229

##### Notes

Morpho-molecular identification (GenBank ID: PQ458160; PQ458161; PQ458162). First record for Lithuania (Bubinas and Jagminienė 2001).

#### Orthocladiinae

Kieffer, 1911

C041BB29-F9C4-5EE0-A434-37EEFDD238CF

#### Acricotopus

Kieffer, 1921

F0C4434B-5926-5BB1-B115-A9B27B4E76B8

#### Acricotopus lucens

(Zetterstedt, 1850)

EC55B117-1518-5EA9-B1A2-D926301E2AF1

##### Notes

Morpho-molecular identification (GenBank ID: PQ458072). New record for Lithuania.

#### Brillia

Kieffer, 1913

B7387E8C-8620-57E4-966F-32CF7F4B513A

#### Brillia sp.

2AFB6040-E969-5C14-9201-FC0F07A04037

##### Notes

Morpho identification. First record for Lithuania (Pliūraitė 2001a).

#### Chaetocladius

Kieffer, 1911

3E6F18EF-EF59-5D5F-BFA1-90F02A56D9F9

#### Chaetocladius (Chaetocladius) piger

(Goetghebuer, 1913)

E29A4F43-35B8-5311-B7EA-6E3E5E44F1E0

##### Notes

Morpho identification. New record for Lithuania.

#### Corynoneura

Winnertz, 1846

37BB3F67-CE62-5AC4-9840-F54CA563A5E7

#### Corynoneura sp.

31113E99-2A12-5550-B81E-DB42A5FD796F

##### Notes

Morpho identification. First record for Lithuania (Grigelis 1999).

#### Cricotopus

van der Wulp, 1874

DCD3536A-F260-5CD1-AB36-4A969889FDF7

#### Cricotopus (Cricotopus) bicinctus

(Meigen, 1818)

AD6F73BD-A2FC-5823-B888-690360F9FA8F

##### Notes

Morpho-molecular identification (GenBank ID: PQ458098). First record for Lithuania (Grigelis 1999).

#### Cricotopus (Cricotopus) cylindraceus

(Kieffer, 1908)

E18FA2BE-9189-5E61-9850-EF1BFD9CE877

##### Notes

Morpho identification. New record for Lithuania.

#### Cricotopus (Cricotopus) festivellus

(Kieffer, 1906)

AA5DC6A6-D693-522E-9EA0-75BE397B1CC0

##### Notes

Morpho identification. New record for Lithuania.

#### Cricotopus (Isocladius) perniger

(Zetterstedt, 1850)

317CA56A-F391-561E-AD52-22842E5A17A5

##### Notes

Morpho identification. New record for Lithuania.

#### Cricotopus sp.

431BD327-A268-5B8D-8956-04AAE618E700

##### Notes

Morpho-molecular identification (GenBank ID: PQ458100). First record for Lithuania (Grigelis 1999).

#### Cricotopus (Isocladius) sylvestris

Fabricius, 1794

7B0D7709-EE7C-5B86-AD7B-60862301DE32

##### Notes

Morpho-molecular identification (GenBank ID: PQ458099). First record for Lithuania (Grigelis 1999).

#### Epoicocladius

Sulc and Zavřel, 1924

238AA6F0-2EEF-57B9-9126-9E81C49913C3

#### Epoicocladius ephemerae

(Kieffer, 1924)

7536CFEF-E50D-5886-B232-AFA2D7E61085

##### Notes

Morpho identification. First record for Lithuania (Virbickas and Pliūraitė 2002).

#### Eukiefferiella

Thienemann, 1926

81E47FE0-FA62-5613-9119-04626E50993F

#### Eukiefferiella sp.

0A06F9B2-EFCC-5AE5-8B40-315FBD8B1FED

##### Notes

Morpho identification. First record for Lithuania (Pliūraitė 1999).

#### Heterotrissocladius

Spärck, 1923

ED773001-5D74-590C-8248-7DC9279E6262

#### Heterotrissocladius marcidus

(Walker, 1856)

6EBA0F2C-08A8-5B04-AB2A-0680F8C646C4

##### Notes

Morpho identification. New record for Lithuania.

#### Limnophyes

Eaton, 1875

B5094EE7-5F4D-5D8E-B921-405B2CECB0C2

#### Limnophyes minimus

(Meigen, 1818)

13E9B5D6-DEEB-5A9E-8EE9-C08DEB5CDE0D

##### Notes

Morpho identification. First record for Lithuania (Grigelis 1999).

#### Metriocnemus

van der Wulp, 1874

C46F6A04-88C5-5432-BBEC-D7371E4BF089

#### Metriocnemus (Metriocnemus) eurynotus

(Holmgren, 1883)

165B45FD-C673-5A49-B7F1-7852EB656EE8

##### Notes

Morpho identification. First record for Lithuania (Sæther and Spies 2005).

#### Nanocladius

Kieffer, 1913

5360B556-26E0-5419-80F4-343BC5DB275A

#### Nanocladius (Nanocladius) dichromus

(Kieffer, 1906)

382597CE-9288-5E4D-9A8A-87865B5B17AB

##### Notes

Morpho-molecular identification (GenBank ID: PQ458132; PQ458133). First record for Lithuania (Grigelis 1999).

#### Orthocladius

van der Wulp, 1874

4ABCCCF8-EBB7-56A8-9062-B6E184A1342C

#### Orthocladius (Orthocladius) decoratus

(Holmgren, 1869)

A49CBD24-A73D-5CC4-906A-223E6B0ABDEC

##### Notes

Morpho identification. New record for Lithuania.

#### Orthocladius (Orthocladius) oblidens

(Walker, 1856)

36260112-9A6B-5505-90D4-F2C45D286FDB

##### Notes

Morpho-molecular identification (GenBank ID: PQ458135; PQ458136; PQ458137). New record for Lithuania.

#### Orthocladius (Orthocladius) rubicundus

(Meigen, 1818)

82B38466-F5F9-5969-BA96-F775FE8D00CB

##### Notes

Morpho-molecular identification (GenBank ID: PQ458138). First record for Lithuania (Grigelis 1999).

#### Paracladius

Hirvenoja, 1973

E6829034-0492-5F48-8EFF-1D8077466196

#### Paracladius conversus

(Walker, 1856)

9883D9EF-3F69-5069-BFB5-1912F649FE64

##### Notes

Morpho identification. First record for Lithuania (Sæther and Spies 2005).

#### Parakiefferiella

Thienemann, 1936

D75E5098-96AC-5BC5-A550-46937AD1946E

#### Parakiefferiella sp.

969257F4-0985-5A33-A537-7CAD9FB96486

##### Notes

Morpho identification. First record for Lithuania (Ashe and Cranston 1990).

#### Parametriocnemus

Goetghebuer, 1932

1872CDB4-E4AD-59E1-808A-FC2A2A6C0CD0

#### Parametriocnemus sp.

A73369B3-D170-5215-8D2C-AEFE00A704C3

##### Notes

Morpho identification. New record for Lithuania.

#### Paraphaenocladius

Thienemann, 1924

4A7FDF4F-CEE5-5F71-9472-23DBC8FB0DF9

#### Paraphaenocladius sp.

8ABF2A5A-03F3-5A72-85EA-982ECE3F6782

##### Notes

Morpho-molecular identification (GenBank ID: PQ458064; PQ458065). New record for Lithuania.

#### Psectrocladius

Kieffer, 1906

19260898-2B94-5B28-8345-E53D5A0F44D9

#### Psectrocladius (Psectrocladius) limbatellus

(Holmgren, 1869)

72B394EC-4B22-51FB-A719-D352835C732B

##### Notes

Morpho identification. New record for Lithuania.

#### Psectrocladius (Psectrocladius) psilopterus

Thienemann, 1906)

5C9E76B1-C832-59F8-951F-6A2FA1B6DF08

##### Notes

Morpho identification. First record for Lithuania (Grigelis 1999).

#### Psectrocladius (Psectrocladius) sordidellus

(Zetterstedt, 1838)

6BA82BC4-D389-528B-8D70-C675EB31995B

##### Notes

Morpho identification. First record for Lithuania (Grigelis 1999).

#### Rheocricotopus

Brundin, 1956

FA43AAC0-B739-5B5E-9922-09240C681FE3

#### Rheocricotopus (Rheocricotopus) fuscipes

(Kieffer, 1909)

1CFD3634-7B93-5F93-BFB3-733A59F01923

##### Notes

Morpho identification. New record for Lithuania.

#### Synorthocladius

Thienemann, 1935

38C27433-3B3D-5A58-AE1D-5442524AB860

#### Synorthocladius semivirens

(Kieffer, 1909)

E41AE9E6-7B27-58F5-A3E2-253F7C419683

##### Notes

Morpho identification. First record for Lithuania (Grigelis 1999).

#### Thienemanniella

Kieffer, 1911

CE2B19FB-7D60-581A-969C-70BE3D59C74E

#### Thienemanniella sp.

A5EA6AC9-BC2A-511A-8F99-ED94B29DB2A2

##### Notes

Morpho identification. New record for Lithuania.

#### Zalutschia

Lipina, 1939

6D6658C9-D6B9-5017-AFCF-1397AE807E49

#### Zalutschia sp.

62C9CEAE-8CD3-54F6-9E25-2808229CBA23

##### Notes

Morpho identification. First record for Lithuania (Pliūraitė 1999).

#### Chironominae

Newman, 1834

1D9A5DE4-566F-51C0-B45F-627B0C9F5FA5

#### Chironomini

Newman, 1834

CA944670-5B61-5563-AAA6-167727AB0A02

#### Chironomus

Meigen, 1803

64AE1375-46BA-5562-BA23-6F1BE63FA899

#### Chironomus (Chironomus) acidophilus

Keyl, 1960

35AA815B-C766-5141-8C77-3BC875F0099D

##### Notes

Morpho-molecular identification (GenBank ID: PQ458075). New record for Lithuania.

#### Chironomus (Chironomus) cingulatus

Meigen, 1830

4A5EAD9A-DC5B-5A74-BB8A-854E6F55B4DD

##### Notes

Morpho-molecular identification (GenBank ID: PQ458076; PQ458077; PQ458078; PQ458079). New record for Lithuania.

#### Chironomus (Chironomus) curabilis

Belyanina, Sigareva and Loginova, 1990

FFBCA014-BD52-535C-A9E5-A3E64A030808

##### Notes

Morpho-molecular identification (GenBank ID: PQ458080; PQ458081). New record for Lithuania.

#### Chironomus (Chironomus) melanescens

Keyl, 1961

A6E8CEBB-9C24-53D1-99C4-78CA787FF642

##### Notes

Morpho-molecular identification (GenBank ID: PQ458082). New record for Lithuania.

#### Chironomus (Chironomus) melanotus

Keyl, 1961

CC50177E-C7E5-5B5D-9AD5-E46E7B73E452

##### Notes

Morpho-molecular identification (GenBank ID: PQ458083). New record for Lithuania.

#### Chironomus (Chironomus) pallidivittatus

Malloch, 1915

6C249142-92B2-5BA9-A6C4-DB56E5287B43

##### Notes

Morpho-molecular identification (GenBank ID: PQ458085). New record for Lithuania.

#### Chironomus (Chironomus) piger

Strenzke, 1959

1F821DCA-E1C3-5167-A75A-2C02AD012048

##### Notes

Morpho-molecular identification (GenBank ID: PQ458086; PQ458087). New record for Lithuania.

#### Chironomus (Chironomus) plumosus

(Linnaeus, 1758)

CE41E406-657E-547D-AB15-3FFE079BBBF6

##### Notes

Morpho identification. First record for Lithuania (Grigelis et al. 1981).

#### Chironomus (Chironomus) pseudothummi

Strenzke, 1959

AAC38AE8-9962-5FF9-9181-D4114367217D

##### Notes

Morpho-molecular identification (GenBank ID: PQ458084; PQ458088). New record for Lithuania.

#### Chironomus (Chironomus) riparius

Meigen, 1804

3BF12255-575E-5B39-84B8-B88202007B90

##### Notes

Morpho identification. First record for Lithuania (Grigelis 1999).

#### Chironomus (Chironomus) salinarius

Kieffer, 1915

AB6D3702-DEED-5EDA-BAE7-AA41D289756A

##### Notes

Morpho identification. First record for Lithuania (Grigelis 1999).

#### Cladopelma

Kieffer, 1921

5E4C1138-375D-5497-A384-12034CF660BA

#### Cladopelma sp.

6257E939-125A-5F85-9525-585B4B3AF5CE

##### Notes

Morpho identification. First record for Lithuania (Ashe and Cranston 1990).

#### Cryptochironomus

Kieffer, 1918

7474720A-85D4-577A-9E7A-4B3EB4474ADC

#### Cryptochironomus albofasciatus

(Staeger, 1839)

13EC11F4-BC0A-52E2-9E76-7E5199D1681B

##### Notes

Morpho-molecular identification (GenBank ID: PQ458101; PQ458102). New record for Lithuania.

#### Cryptochironomus obreptans

(Walker, 1856)

541EE618-1922-5D24-96E1-C86D3A82D97A

##### Notes

Morpho-molecular identification (GenBank ID: PQ458103). New record for Lithuania.

#### Cryptochironomus rostratus

Kieffer, 1921

D57D88CC-7410-5402-B84F-18C1F1F25A29

##### Notes

Morpho-molecular identification (GenBank ID: PQ458104). New record for Lithuania.

#### Cryptotendipes

Beck and Beck, 1969

49413F9B-BD6B-5A33-8741-6CC608C89572

#### Cryptotendipes sp.

EE0F960B-45BE-5F9F-9F21-7FBEE0B53494

##### Notes

Morpho identification. First record for Lithuania (Sæther and Spies 2005).

#### Demicryptochironomus

Lenz, 1941

56DC47A6-90C4-5B1A-9034-2592DA35C7CB

#### Demicryptochironomus (Demicryptochironomus) vulneratus

(Zetterstedt, 1838)

59DF2FD5-12A6-5CC0-8E59-85F1B80CE8EA

##### Notes

Morpho-molecular identification (GenBank ID: PQ458105; PQ458106). First record for Lithuania (Ashe and Cranston 1990).

#### Dicrotendipes

Kieffer, 1913

EA91D811-43D7-5B19-9D8B-BD99975FD66A

#### Dicrotendipes nervosus

(Staeger, 1839)

2D390F28-DC82-52B1-9CC5-FB999A3588A0

##### Notes

Morpho identification. First record for Lithuania (Grigelis 1999).

#### Dicrotendipes tritomus

Thienemann & Kieffer, 1916

9CBD93C9-1466-58A1-90BF-2547ADFA3CE6

##### Notes

Morpho-molecular identification (GenBank ID: PQ458107). First record for Lithuania (Grigelis 1999).

#### Einfeldia

Kieffer, 1924

EBE6C9DC-2F0B-5560-84CD-AE7A6AFC6A1E

#### Einfeldia pagana

(Meigen, 1838)

22778DBE-8AAA-5A86-A692-0CC463FF52CD

##### Notes

Morpho-molecular identification (GenBank ID: PQ458108; PQ458109). First record for Lithuania (Grigelis 1999).

#### Endochironomus

Kieffer, 1918

1DC400EA-0BE0-50EB-8992-EACF74DC7C0E

#### Endochironomus albipennis

(Meigen, 1830)

9E8EEC46-B7AA-53D7-8C78-E7FCD21BBA18

##### Notes

Morpho identification. First record for Lithuania (Grigelis 1999).

#### Endochironomus tendens

(Fabricius, 1775)

FCEDB5BD-441F-5E24-929B-CEF2BB594EA2

##### Notes

Morpho-molecular identification (GenBank ID: PQ458110; PQ458111). First record for Lithuania (Grigelis 1999).

#### Glyptotendipes

Kieffer, 1913

88CD620D-C783-5076-9C59-333DC552F3FE

#### Glyptotendipes (Phytotendipes) cauliginellus

(Kieffer, 1913)

FF1C0603-3AFC-5BB5-9C90-3DD5A741767D

##### Notes

Morpho-molecular identification (GenBank ID: PQ458112). First record for Lithuania (Grigelis 1999).

#### Glyptotendipes (Phytotendipes) pallens

(Meigen, 1804)

36149A10-BA4E-525D-838A-DB1A58B2EF3B

##### Notes

Morpho identification. First record for Lithuania (Pliūraitė and Kesminas 2004).

#### Glyptotendipes (Trichotendipes) signatus

(Kieffer, 1909)

9D2B3430-86E8-52F6-8F63-DA962C7D1235

##### Notes

Morpho identification. New record for Lithuania.

#### Harnischia

Kieffer, 1921

1FF6D0C5-6AD2-56E1-AD37-5082F12C6678

#### Harnischia fuscimanus

Kieffer, 1921

582B08F1-8780-5523-9CB6-3849D0C9CD84

##### Notes

Morpho-molecular identification (GenBank ID: PQ458113). First record for Lithuania (Pliūraitė 2001a).

#### Microtendipes

Kieffer, 1915

12E76F32-4D1E-5FF1-87E4-455642A3B845

#### Microtendipes chloris

(Meigen, 1818)

A25D3888-6F07-54C4-A3E2-0D0C7CD5CFDF

##### Notes

Morpho-molecular identification (GenBank ID: PQ458117; PQ458118; PQ458119; PQ458120; PQ458121). First record for Lithuania (Grigelis 1999).

#### Microtendipes pedellus

(De Geer, 1776)

5313C1B6-8D1B-5D39-BC2A-B8660AA16246

##### Notes

Morpho-molecular identification (GenBank ID: PQ458122; PQ458123; PQ458124; PQ458125; PQ458126; PQ458127). First record for Lithuania (Grigelis 1999).

#### Microtendipes rydalensis

(Edwards, 1929)

5B9E18C1-00AF-5BE8-B6A2-D87E213DD71C

##### Notes

Morpho-molecular identification (GenBank ID: PQ458128). New record for Lithuania.

#### Microtendipes sp.

FD666062-87A5-5C8D-A636-36EA4110D9CA

##### Notes

Morpho-molecular identification (GenBank ID: PQ458129). First record for Lithuania (Grigelis 1999).

#### Microtendipes tarsalis

(Walker, 1856)

AF836B53-55F9-5AF6-9E1E-872AB1BCDC1B

##### Notes

Morpho identification. First record for Lithuania (Grigelis 1999).

#### Parachironomus

Lenz, 1921

A21E2D68-01E8-5F5F-8301-9218B90D4984

#### Parachironomus vitiosus

(Goetghebuer, 1921)

1C027F76-0F31-5464-B936-89EED3FAB850

##### Notes

Morpho-molecular identification (GenBank ID: PQ458139; PQ458140; PQ458141). First record for Lithuania (Ashe and Cranston 1990).

#### Paracladopelma

Harnisch, 1923

009874CD-5189-5927-A1EC-1004AA80456C

#### Paracladopelma camptolabis

(Kieffer, 1913)

43F8FE62-4979-53E6-A7E7-3CA433B4D0F3

##### Notes

Morpho identification. First record for Lithuania (Pliūraitė and Kesminas 2004).

#### Paratendipes

Kieffer, 1911

035F99EB-BA82-5DF6-A705-6DD7B45B796A

#### Paratendipes albimanus

(Meigen, 1804)

7D427609-00FD-55F6-905F-CB0C8FF4736B

##### Notes

Morpho-molecular identification (GenBank ID: PQ458142; PQ458143; PQ458144; PQ458145). First record for Lithuania (Grigelis 1999).

#### Polypedilum

Kieffer, 1912

203EC7DF-55EB-56E8-A1E8-0F2B5D7E248F

#### Polypedilum (Uresipedilum) convictum

(Walker, 1856)

2DEE4C7C-F397-50CF-9E76-E3FB0332898A

##### Notes

Morpho-molecular identification (GenBank ID: PQ458146). First record for Lithuania (Grigelis 1999).

#### Polypedilum (Uresipedilum) cultellatum

Goetghebuer, 1931

7D13F44D-84D7-5168-8BEB-386CCEE4EE11

##### Notes

Morpho-molecular identification (GenBank ID: PQ458147). New record for Lithuania.

#### Polypedilum (Polypedilum) nubeculosum

(Meigen, 1804)

443E34EC-AAB4-5030-9355-A77C1FD87E2D

##### Notes

Morpho identification. First record for Lithuania (Ashe and Cranston 1990).

#### Polypedilum (Tripodura) pullum

(Zetterstedt, 1838)

6CDCE518-C43E-5D79-9DA0-55815393377A

##### Notes

Morpho identification. New record for Lithuania.

#### Polypedilum (Tripodura) scalaenum

Schrank, 1803

3B5AD1A7-0322-587C-A473-D2666460B92C

##### Notes

Morpho-molecular identification (GenBank ID: PQ458148; PQ458149). First record for Lithuania (Grigelis 1999).

#### Polypedilum (Pentapedilum) sordens

(Wulp, 1875)

DEC452A0-7D3B-5A6A-9AEB-1FE6B6C3A8E0

##### Notes

Morpho identification. First record for Lithuania (Ashe and Cranston 1990).

#### Polypedilum sp.

0DF6C57B-BE6F-5488-96B9-C4424AD26139

##### Notes

Morpho-molecular identification (GenBank ID: PQ458150). First record for Lithuania (Lapinskaitė 1968).

#### Synendotendipes

Grodhaus, 1987

7FE93FEA-70C7-5C69-B59C-B615721C1685

#### Synendotendipes impar

(Walker, 1856)

3B2AE763-B5D2-517C-BE2A-D3CD3E502282

##### Notes

Morpho-molecular identification (GenBank ID: PQ458166). First record for Lithuania (Pliūraitė 1999).

#### Stenochironomus

Kieffer, 1919

A892C24D-49B7-5C5B-A7FF-72A483031EA0

#### Stenochironomus (Stenochironomus) gibbus

(Fabricius, 1794)

F4E8CEE4-5647-530F-939F-D962EA0400A3

##### Notes

Morpho-molecular identification (GenBank ID: PQ458164; PQ458165). New record for Lithuania.

#### Stictochironomus

Kieffer, 1919

9BDFDD30-E906-55EB-AF01-9E380038FD87

#### Stictochironomus sp.

0644E609-BA44-5F0F-A789-858B9D730EAD

##### Notes

Morpho identification. First record for Lithuania (Grigelis 1999).

#### Tribelos

Townes, 1945

CD8F049E-380E-5D2C-AFEB-DBC8CB9DD2D3

#### Tribelos intextus

(Walker, 1856)

C7B31308-A380-5008-889E-7BFF0F4821EA

##### Notes

Morpho-molecular identification (GenBank ID: PQ458171; PQ458172). First record for Lithuania (Ashe and Cranston 1990).

#### Xenochironomus

Kieffer, 1921

713171A1-32DC-5FFA-B2B8-0F506D54CE61

#### Xenochironomus xenolabis

Kieffer, 1916

63F0771E-AE98-59FA-9737-6D457E688DEB

##### Notes

Morpho identification. First record for Lithuania (Pliūraitė 1999).

#### Tanytarsini

Zavřel, 1917

1E4BE212-D336-53A0-A839-7E8263B7AD26

#### Cladotanytarsus

Kieffer, 1921

32D78B00-01E7-5A2F-88CC-DF7FCB6E2085

#### Cladotanytarsus mancus

(Walker, 1856)

AB2BF645-0D74-557E-BC8F-B90EC7F7603C

##### Notes

Morpho identification. First record for Lithuania (Ashe and Cranston 1990).

#### Micropsectra

Kieffer, 1908

E42E5DEA-3CF7-5ACF-BAC1-5FAEB3B1B191

#### Micropsectra apposita

(Walker, 1856)

3BA4F1B9-3904-5E81-895C-1415AF0778DD

##### Notes

Morpho identification. New record for Lithuania.

#### Micropsectra contracta

Reiss, 1965

E69BB4D7-0A5E-5D05-8DD9-72E216009A0A

##### Notes

Morpho-molecular identification (GenBank ID: PQ458116). First record for Lithuania (Grigelis 1999).

#### Neozavrelia

Goetghebuer, 1941

10D1FE20-E653-5A11-A5DA-9725D8F307F0

#### Neozavrelia sp.

63CDC7A7-9A85-58C4-9784-907EB9385729

##### Notes

Morpho identification. New record for Lithuania.

#### Paratanytarsus

Thienemann and Bause, 1913

07B1B140-8A07-54D1-8037-B35CEF981A8A

#### Paratanytarsus sp.

37132BBD-D824-56E9-90B3-DF67929845EB

##### Notes

Morpho identification. First record for Lithuania (Grigelis 1999).

#### Stempellina

Thienemann and Bause, 1913

77EFA6A6-DBCD-54EE-A0C0-C7B6D0D57ED3

#### Stempellina sp.

C4C8C121-540B-55EE-A097-8456D4EE189E

##### Notes

Morpho identification. First record for Lithuania (Grigelis 1999).

#### Tanytarsus

van der Wulp, 1874

402616D6-7418-5B6D-A103-B9B1BAEC596C

#### Tanytarsus multipunctatus

Brundin, 1947

770AF176-0D3E-55C2-83B5-2F13E31FB179

##### Notes

Morpho-molecular identification (GenBank ID: PQ458168; PQ458169; PQ458170). New record for Lithuania.

#### Virgatanytarsus

Pinder, 1982

5A1A80D7-019D-5E48-B14B-8C4EE4A97DE6

#### Virgatanytarsus sp.

8C383BB6-7717-573F-8AB6-A8609F7221A9

##### Notes

Morpho-molecular identification (GenBank ID: PQ458167). New record for Lithuania.

## Analysis

A grand total of 11,296 non-biting midge specimens, comprising 89 species representing 65 genera and five subfamilies, were gathered from the sampling sites from six streams. Most specimens were identified to the subfamily and genus level, based on both morphological characteristics and analysis of partial COI sequences.

**Figure 3.**
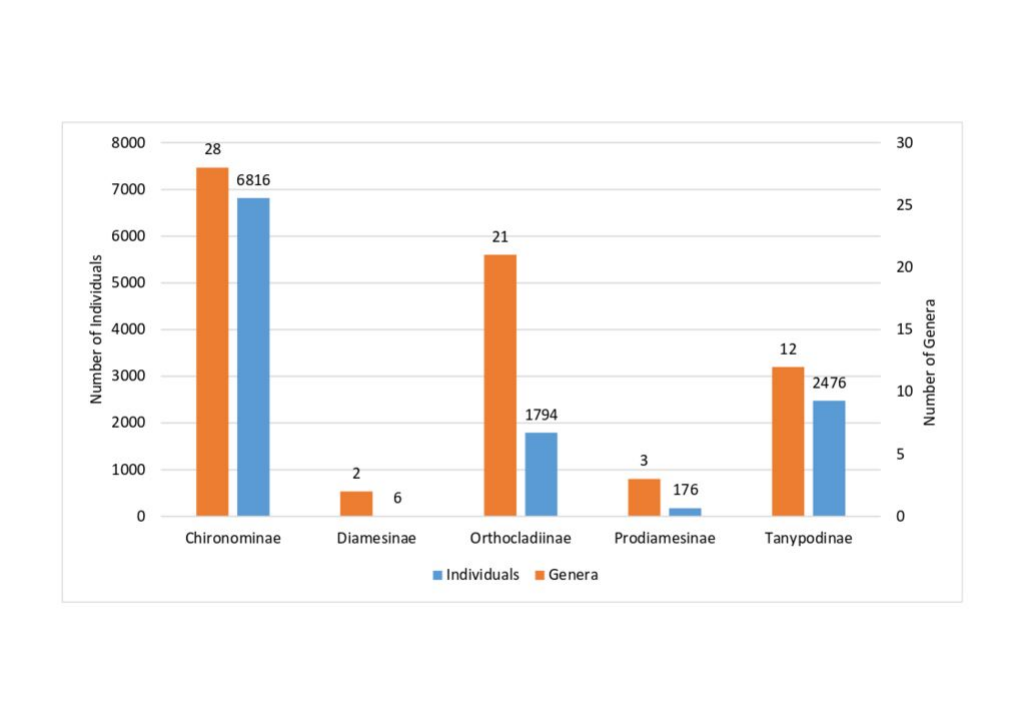
The amount of individuals (blue bars) and the distribution across genera (orange bars) of non-biting midges’ (Chironomidae) subfamilies collected during the study.

The highest richness of chironomids was observed in the subfamily Chironominae, which included 28 genera and 45 species. In contrast, the lowest richness was found in Diamesinae, with two genera and two species (Fig. 3). The Orthocladiinae exhibited a richness of 21 genera and 21 species, while Prodiamesinae showed a richness of 13 genera and 19 species.

In terms of abundance, the subfamilies Chironominae (6816 specimens) and Tanypodinae (2476 specimens) were the most prevalent, together accounting for 82.46% of collected specimens. Conversely, the subfamilies Prodiamesinae and Diamesinae subfamilies had the fewest individuals, with specimens 176 and six specimens, respectively.

**Figure 4.**
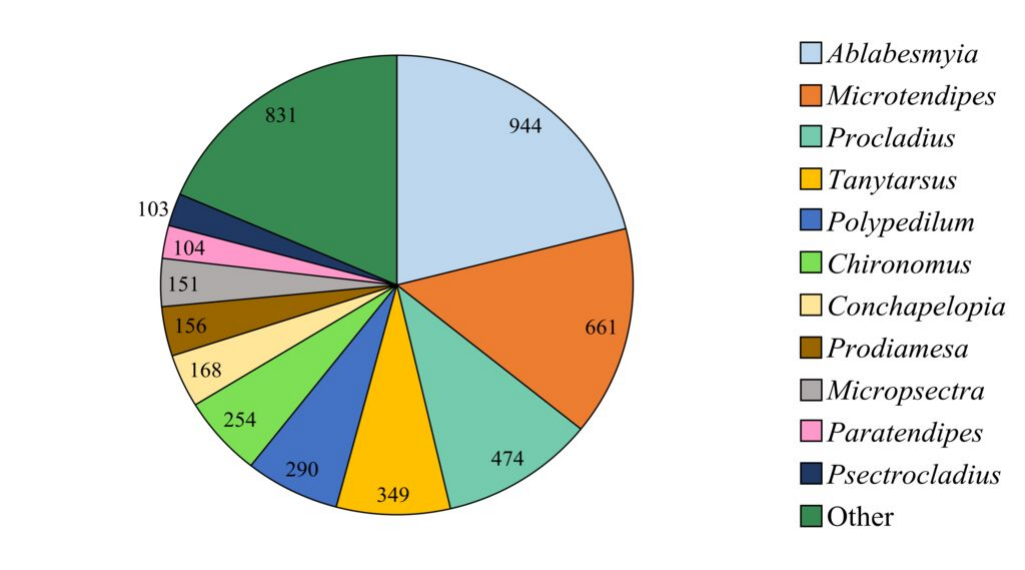
The number of specimens of non-biting midges (Chironomidae) representing each genus after excluding those with less than 100 collected specimens.

Amongst the genera, the most abundant were *Ablabesmyia* Johannsen, 1905 (944 specimens), *Microtendipes* Kieffer, 1915 (661 specimens), *Procladius* Skuse, 1889 (474 specimens), *Tanytarsus* van der Wulp, 1874 (349 specimens), *Polypedilum* Kieffer, 1912 (290 specimens), *Chironomus* Meigen, 1803 (254 specimens), *Conchapelopia* Fittkau, 1957 (168 specimens), *Prodiamesa* Kieffer, 1906 (156 specimens), *Micropsectra* Kieffer, 1908 (151 specimens), *Paratendipes* Kieffer, 1911 (104 specimens) and *Psectrocladius* Kieffer, 1906 (103 specimens) (Fig. 4).

The Table below lists the subfamilies, genera and the abundance of specimens in each of the studied streams: Dubinga, Kiauna, Luknelė, Plaštaka, Skerdyksna and Šešuola in 2021 and 2022, spanning from May to September (Table 2).

**Figure 5.**
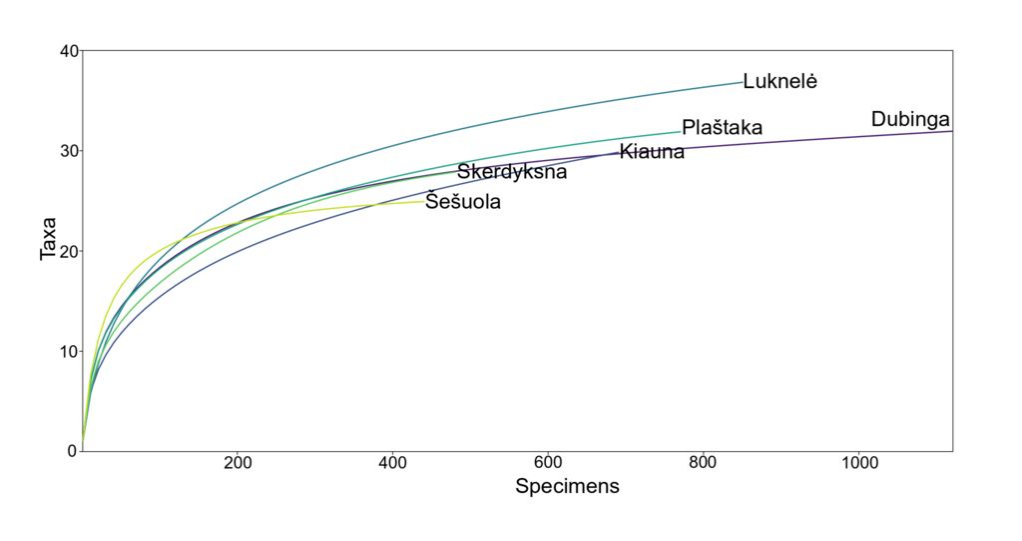
Sample-based genera accumulation (rarefaction) curves, with 95% confidence intervals, of the Chironomidae genera’ richness collected in six streams.

The genera richness of non-biting midges reached an asymptote in all the streams studied, except for the Kiauna stream, suggesting that additional genera may still be discovered (Fig. 5).

**Figure 6.**
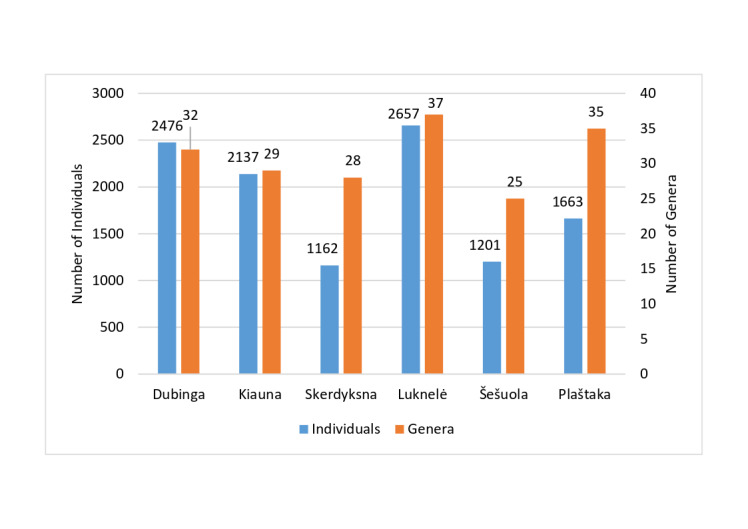
Number of genera (orange bars) and specimens (blue bars) of non-biting midges (Chironomidae) in all streams researched.

**Figure 7.**
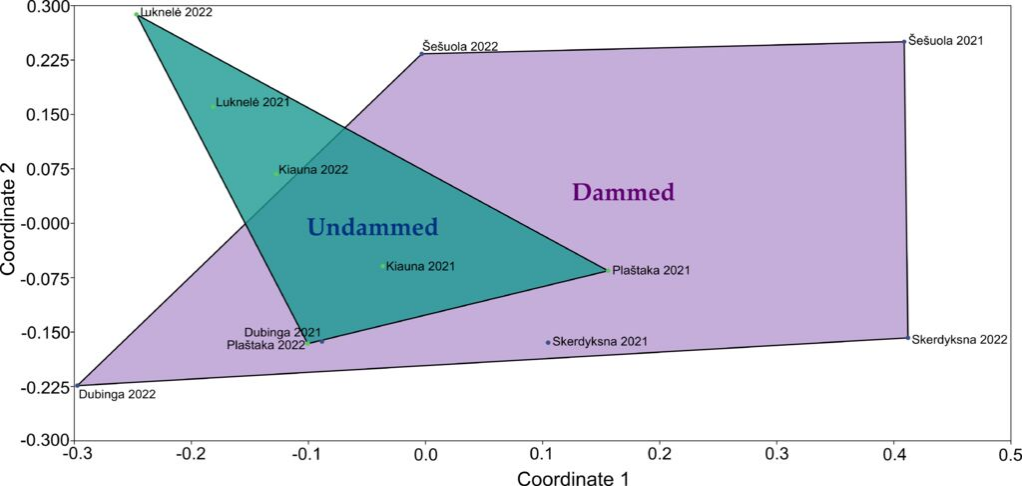
Non-metric multidimensional scaling (NMDS) ordination (stress = 0.167) representing Chironomidae assemblages in dammed (purple) and undammed (blue) streams.

Amongst the undammed streams, the Luknelė had the highest abundance of individuals and the greatest number of genera identified. It also emerged as a stream with the highest abundance and diversity of chironomids in terms of genera across all the streams studied. In contrast, the Šešuola stream had the lowest number of individuals, the least diversity of genera across all the studied streams and was the only stream that was dammed twice (Fig. 6). Since three of the streams studied were dammed and three were undammed, NMDS analysis was conducted to compare them based on chironomid assemblages. The visual analysis showed that the genera composition of chironomids in dammed and undammed streams partially overlapped (Fig. 7).

## Discussion

Our study represents the first comprehensive investigation into the Chironomidae family in Lithuania. Despite studying only six streams with similar characteristics within one region of Lithuania, the gathered chironomids material encompasses over 50% of all known non-biting midges species in the country. However, given the limited scope of our research and its outcomes, it is apparent that the findings may not entirely reflect the actual state of Chironomidae in Lithuania.

The prevalence of subfamilies in terms of genera and number of individuals reveals notable trends, aligning closely with observations made in Croatia by Čerba et al. (2020), who investigated chironomid fauna across diverse freshwater habitats. Their study, like ours, underscores the dominance of the Chironominae subfamily, while also noting a relatively lower richness within the Prodiamesinae subfamily.

According to the latest data on Lithuanian non-biting midge species (Pakalniškis et al. 2006, Ruginis 2007, Móra and Kovács 2009), our research contributes significantly to the understanding of the biodiversity within the Chironomidae family in Lithuania. The discovery of several species and genera new to Lithuania underscores the richness of the region's chironomid fauna and highlights the potential for further entomological exploration. By identifying a substantial proportion of the known Orthocladiinae genera and species, our study sheds light on the ecological complexity and diversity of this subfamily. The identification of new species and genera within the Chironominae and Tanypodinae subfamilies further enhances the existing taxonomic knowledge and provides a valuable foundation for future ecological and environmental studies. These findings not only expand the taxonomic records, but also offer insights into the distribution and ecological roles of these subfamilies in Lithuanian freshwater ecosystems.

Considering the research on Chironomidae conducted in neighbouring countries, it becomes evident that the faunistic knowledge of non-biting midges in Lithuania is relatively limited. In Europe, there are over 190 genera and more than 1260 species of Chironomidae (Paasivirta 2014, Serra et al. 2016, Serra et al. 2017). Germany, in particular, is known for extensive Chironomidae research, with significant efforts concentrated in the Land of Brandenburg (Orendt 2018). While major studies have been conducted in Brandenburg, research has also been carried out in other regions of Germany (Orendt 2000, Brunke 2004, Orendt et al. 2014). Currently, over 165 genera and 780 species of Chironomidae are known in Germany, although there is still a need for further investigation (Chimeno et al. 2022, Chimeno et al. 2023).

Poland has also made significant contributions to Chironomidae research, with studies covering diversity, ecology, biology and other related areas (Płóciennik 2009, Płóciennik et al. 2015, Płóciennik et al. 2018, Leszczyńska et al. 2019, Pleskot et al. 2019, Głowacki et al. 2023). More than 420 species of Chironomidae are known in Poland (Płóciennik 2009). Ukraine, another country in the region where research on Chironomidae has been conducted since the early 20^th^ century, still faces a significant lack of comprehensive study (Baranov 2011). Despite numerous surveys and the documentation of over 300 Chironomidae species, recent investigations have identified 40 additional species and one new genus, suggesting that the research remains incomplete (Baranov 2011, Bitušík et al. 2024). In the Baltic States of Latvia and Estonia, the level of faunistic knowledge of Chironomidae is similar to Lithuania, with a relatively low number of known species (Spuņģis and Kalniņš 2003). This indicates a general trend of limited research in the region regarding non-biting midges.

According to data from Finland, their national Chironomidae assemblage is extensively researched, with over 780 species of non-biting midges currently documented. However, the dynamic nature of changing climate is impacting the diversity, leading to fluctuations in species composition, with several chironomids already listed on the Red List of Finnish Species (Paasivirta 2014, Engels et al. 2019, Hyvärinen et al. 2019). In Sweden, approximately 900 species of Chironomidae are known, which accounts for more than 70% of the total number of Chironomidae species documented in Europe and the country is one of the regional leaders in the research of this group (SLU Swedish Species Information Centre 2024). Norway is another country in the region actively involved in Chironomidae research, with ongoing studies conducted even in challenging environments such as Svalbard and Jan Mayen. More than 70 species of chironomids are currently known in these areas, contributing to a total of over 650 species of Chironomidae documented in Norway (Elven and Søli 2016, Stur and Ekrem 2020).

Based on the rarefaction results analysis, we can conclude that the detection of non-biting midges in the six streams was effective. Although the NMDS analysis revealed significant overlap in genus composition between dammed and non-dammed streams, this does not imply that dams have no impact on Chironomidae diversity and ecology. In conclusion, the understanding of Chironomidae diversity in Lithuania is still evolving. By leveraging the insights gleaned from neighbouring countries, there is the urgent need for Lithuania to continue its research efforts. This should extend beyond the borders of our country, encompassing regional and global initiatives aimed at conserving biodiversity and grappling with the challenges posed by environmental shifts on non-biting midge communities and populations. It would not only enrich the scientific landscape of Lithuania, but also contribute meaningfully to the collective endeavour of safeguarding our natural heritage for future generations.

## Figures and Tables

**Table 1.** List of the six researched streams along with pertinent information.

Sub-basin	Stream	Length (km)	Discharge (m^3^/s)	Catchment area (km^2^)	Dammed	Coordinates of sampling sites			
Žeimena	Dubinga	18.1	4.01	405.9	Yes	1 55°00'51.1"N, 25°38'16.4"E	2 55°00'56.4"N, 25°42'14.4"E	3 54°59'32.9"N, 25°45'16.6"E	4 54°59'27.2"N, 25°45'32.8"E
Žeimena	Skerdyksna	13.9	0.50	42.2	Yes	5 54°56'44.7"N, 25°50'37.2"E	6 54°56'50.9"N, 25°45'35.6"E	7 54°56'47.5"N, 25°45'29.4"E	8 54°57'08.5"N, 25°43'18.7"E
Žeimena	Luknelė	13.6	0.54	45.0	No	9 55°12'49.1"N, 25°56'32.1"E	10 55°13'22.0"N, 25°52'04.6"E	11 55°14'20.9"N, 25°51'14.6"E	12 55°15'00.0"N, 25°50'22.5"E
Žeimena	Kiauna	17.9	2.95	308.7	No	13 55°14'48.5"N, 25°58'14.5"E	14 55°15'50.5"N, 25°56'28.6"E	15 55°17'44.4"N, 25°53'38.0"E	16 55°18'29.6"N, 25°53'12.9"E
Šventoji	Plaštaka	18.1	0.82	88.3	No	17 55°18′29.12″N, 25°1′51.20″E	18 55°18′50.94″N, 25°3′41.15″E	19 55°20′9.35″N, 25°2′11.69″E	20 55°16′40.62″N, 24°59′10.82″E
Šventoji	Šešuola	15.6	0.65	91.7	Yes	21 55°15'10.2"N, 24°58'40.9"E	22 55°15'14.9"N, 24°58'44.0"E	23 55°10'56.5"N, 24°56'28.0"E	24 55°11'03.7"N, 24°56'33.3"E

**Table 2.** List of non-biting midges (Chironomidae) sub-families and genera and their abundance collected of six streams in Lithuania, 2021 to 2022: Dubinga, Kiauna, Luknelė, Plaštaka, Skerdyksna and Šešuola.

Sub-family	Genera	Dubinga	Kiauna	Luknelė	Plaštaka	Skerdyksna	Šešuola
**Tanypodinae**	*Anatopynia*		1	1			
	*Clinotanypus*	5	1	4	13	47	10
	*Apsectrotanypus*			10			
	*Macropelopia*		1	6	2		
	*Psectrotanypus*				4		
	*Ablabesmyia*	119	206	412	75	38	94
	*Conchapelopia*	4	39	73	10	16	26
	*Krenopelopia*			2			
	*Larsia*		4	2			
	*Zavrelimyia*			5		1	
	*Procladius*	84	68	54	81	153	34
	*Tanypus*		2		2		4
**Diamesinae**	*Potthastia*	4					
	*Pseudodiamesa*			1		1	
**Prodiamesinae**	*Monodiamesa*	10		1	2		
	*Odontomesa*				7		
	*Prodiamesa*	69	41	13		3	30
**Orthocladiinae**	*Acricotopus*				23		
	*Brillia*	1					
	*Chaetocladius*				1	1	
	*Corynoneura*	23	9	15	14	5	17
	*Cricotopus*	16		2		4	15
	*Epoicocladius*	3		1			
	*Eukiefferiella*		1		2		
	*Heterotrissocladius*			4			
	*Limnophyes*			1			
	*Metriocnemus*				1		
	*Nanocladius*					2	
	*Orthocladius*		4	5			64
	*Paracladius*		3		1		1
	*Parakiefferiella*	15	9	7			3
	*Parametriocnemus*				3		
	*Paraphaenocladius*			58			
	*Psectrocladius*	91	3	5	2	2	
	*Rheocricotopus*			19			
	*Synorthocladius*	5			2		1
	*Thienemanniella*			1	1		
	*Zalutschia*	2		7			
**Chironominae**	*Chironomus*	38	29		85	59	12
	*Cladopelma*		2				
	*Cryptochironomus*	8	4	4	6	2	
	*Cryptotendipes*				4	1	
	*Demicryptochironomus*	1		15			
	*Dicrotendipes*					3	3
	*Einfeldia*				1	1	17
	*Endochironomus*	2	5		9	2	8
	*Glyptotendipes*	14					2
	*Harnischia*			2	10		
	*Microtendipes*	238	152	39	158	63	10
	*Parachironomus*					19	15
	*Paracladopelma*	1			1	5	
	*Paratendipes*	3	8		60	28	5
	*Polypedilum*	167	10	17	58	4	21
	*Synendotendipes*	9	1	1		3	5
	*Stenochironomus*	8	1				
	*Stictochironomus*		1	2		2	
	*Tribelos*	14					
	*Xenochironomus*			2			
	*Cladotanytarsus*	5	11	27	13		
	*Micropsectra*	69		13	32	3	34
	*Neozavrelia*	7					
	*Paratanytarsus*	1	1	1		3	3
	*Stempellina*		1				
	*Tanytarsus*	97	82	37	101	22	21
	*Virgatanytarsus*	16					

## References

[B11732756] Andersen T., Cranston P., Elper J. (2013). The larvae of Chironomidae (Diptera) of the Holarctic Region – Keys and diagnoses..

[B11743049] Ashe P., Cranston P. S., Soos A., Papp L. (1990). Catalogue of Palaearctic Diptera.

[B12191497] Baranov V. A. (2011). Попередній анотований список комарів-дзвінців (Diptera, Chironomidae) України. [A Preliminary Annotated Cheklist of Non-Biting Midges (Diptera, Chironomidae) of Ukraine]. Ukrainska Entomofaunistyka [Internet].

[B12191506] Bitušík Peter, Novikmec Milan, Svitok Marek, Hamerlík Ladislav (2024). New faunistic records of chironomids and phantom midges (Diptera, Chironomidae and Chaoboridae) from Ukraine indicate recent climatic refugia in the Eastern Carpathians. ZooKeys.

[B11732936] Brunke M., Pusch M., Wilczek S., Schwartz R., Kozerski HP., Fischer H., Brunke M., Engelhardt C., Shukhodolov A., Schulz M. (2004). Die Elbe – Gewässerökologische Bedeutung von Flussbettstrukturen..

[B11743183] Bubinas A., Jagminienė I. (2001). Bioindication of ecotoxity according to community structure of macrozoobenthic fauna. Acta Zoologica Lituanica.

[B11732963] Čerba D., Koh M., Ergović V., Mihaljević Z., Milošević D., Hamerlík L. (2020). Chironomidae (Diptera) of Croatia with notes on the diversity and distribution in various habitat types. Zootaxa.

[B12187997] Chimeno Caroline, Hausmann Axel, Schmidt Stefan, Raupach Michael J., Doczkal Dieter, Baranov Viktor, Hübner Jeremy, Höcherl Amelie, Albrecht Rosa, Jaschhof Mathias, Haszprunar Gerhard, Hebert Paul D. N. (2022). Peering into the Darkness: DNA Barcoding Reveals Surprisingly High Diversity of Unknown Species of Diptera (Insecta) in Germany. Insects.

[B12188014] Chimeno Caroline, Rulik Björn, Manfrin Alessandro, Kalinkat Gregor, Hölker Franz, Baranov Viktor (2023). Facing the infinity: tackling large samples of challenging Chironomidae (Diptera) with an integrative approach. PeerJ.

[B11733057] Cortelezzi Agustina, Simoy María V., Siri Augusto, Donato Mariano, Cepeda Rosana E., Marinelli Claudia B., Berkunsky Igor (2020). New insights on bioindicator value of Chironomids by using occupancy modelling. Ecological Indicators.

[B11733078] Dantas GPS., Amat E., Hamada N. (2024). New species and record of Diplosmittia Sæther, 1981 (Diptera: Chironomidae, Orthocladiinae) from Brazil and Colombia. Zootaxa.

[B11733123] Elven Hallvard, Søli Geir (2016). Kunnskapsstatus for artsmangfoldet i Norge 2015.. Artsdatabanken, Norge..

[B11733132] Engels Stefan, Medeiros Andrew S., Axford Yarrow, Brooks Stephen J., Heiri Oliver, Luoto Tomi P., Nazarova Larisa, Porinchu David F., Quinlan Roberto, Self Angela E. (2019). Temperature change as a driver of spatial patterns and long‐term trends in chironomid (Insecta: Diptera) diversity. Global Change Biology.

[B11733176] Epler J. H. (2001). Identification manual for the larval Chironomidae (Diptera) of North and South Carolina.

[B11733234] Farias R L, Carvalho L K, Medeiros E S F (2012). Distribution of Chironomidae in a Semiarid Intermittent River of Brazil. Neotropical Entomology.

[B11733261] Folmer O., Black M., Hoeh W., Lutz R., Vrijenhoek R. (1994). DNA primers for amplification of mitochondrial cytochrome c oxidase subunit I from diverse metazoan invertebrates. Molecular Marine Biology and Biotechnology.

[B11733298] Gailiušis B., Jablonskis J., Kovalenkovienė M. (2001). Lietuvos upės: hidrografija ir nuotėkis.

[B11743226] Gasiūnas I., institutas Lietuvos TSR Mokslų akademija Biologijos (1959). The Kuršių Marios Gulf.

[B11733346] Głowacki Łukasz, Leszczyńska Joanna, Grzybkowska Maria, Pyrzanowski Kacper, Dukowska Małgorzata, Przybylski Mirosław (2023). Determinants of chironomid species richness in mid-European temperate rivers – Environmental factors, regional influences, diversity, and seasons. Ecological Indicators.

[B11944994] Gotelli N. J., Colwell R. K. (2001). Quantifying biodiversity: Procedures and pitfalls in the measurement and comparison of species richness. Ecology Letters.

[B11743014] Grigelis A., Nainaitė O., Cukerzis J., Šeštokas J., Giniūnas K., Balevičius K., Butkevičius I. (1981). Lietuvos TSR nacionalinis parkas..

[B11743036] Grigelis A., Volskis R., Kangur M. (1999). Hydrobiological Research in the Baltic Countries. Part I. Rivers and Lakes..

[B11733375] Hall T. A. (1999). BioEdit: a user-friendly biological sequence alignment editor and analysis program for Windows 95/98/NT. Oxford University Press.

[B11944985] Hammer Ø., Harper D. A.T., Ryan P. D. (2001). Past: Paleontological Statistics Software Package for Education and Data Analysis. Palaeontologia Electronica.

[B11733402] Hyvärinen E., Juslén A., Kemppainen E., Uddström A., Liukko U. M. (2019). The 2019 Red List of Finnish Species.

[B11733411] Ivković Marija, Dorić Valentina, Baranov Viktor, Mihaljević Zlatko, Kolcsár Levente-Péter, Kvifte Gunnar Mikalsen, Nerudova Jana, Pont Adrian C. (2020). Checklist of aquatic Diptera (Insecta) of Plitvice Lakes National Park, Croatia, a UNESCO world heritage site. ZooKeys.

[B11733424] Jablonskis J., Kovalenkovienė M., Tomkevičienė A. (2007). Lietuvos upių ir upelių vagų tinklas. Annales Geographicae.

[B11733433] Kilkus K., Stonevičius E. (2011). Lietuvos vandenų geografija.

[B11743277] Lapinskaitė J. (1968). Benthonic fauna of lake Žuvintas.

[B11733622] Lencioni Valeria, Marziali Laura, Rossaro Bruno (2012). Chironomids as bioindicators of environmental quality in mountain springs. Freshwater Science.

[B11733441] Leszczyńska Joanna, Grzybkowska Maria, Głowacki Łukasz, Dukowska Małgorzata (2019). Environmental variables influencing chironomid assemblages (Diptera: Chironomidae) in lowland rivers of central Poland. Environmental Entomology.

[B11733450] Letovsky Erin, Myers Ian E, Canepa Alexandra, McCabe Declan J (2012). Differences between kick sampling techniques and short-term Hester-Dendy sampling for stream macroinvertebrates. BIOS.

[B11733637] ministerija Lietuvos Respublikos aplinkos https://uetk.biip.lt/.

[B11733459] Móra A., Kovács T. (2009). Non-biting midges species (Diptera: Chironomidae). New and Rare for Lithuania Insect Species.

[B11733647] Medicine National Library of http://blast.ncbi.nlm.nih.gov/.

[B11733468] Orendt Claus (2000). The chironomid communities of woodland springs and spring brooks, severely endangered and impacted ecosystems in a lowland region of eastern Germany (Diptera: Chironomidae). Journal of Insect Conservation.

[B11733655] Orendt C., Dettinger-Klemm A., Spies M. E. (2011). Bestimmungsschlüssel für die Larven der Chironomidae (Diptera) der Brackgewässer Deutschlands und angrenzender Gebiete.

[B11733489] Orendt C., Garcia X. F., Janecek B. F., Michiels S., Otto C. J., Müller R. (2014). Faunistic overview of Chironomidae (Insecta: Diptera) in lowland running waters of north-east Germany (Brandenburg) based on 10-year EU-Water Framework Directive monitoring programme. Lauterbornia.

[B11733479] Orendt Claus (2018). Results of 10 years sampling of Chironomidae from German lowland running waters differing in degradation. Journal of Limnology.

[B11733500] Paasivirta Lauri (2014). Checklist of the family Chironomidae (Diptera) of Finland. ZooKeys.

[B11733509] Pakalniškis S., Bernotienė R., Lutovinovas E., Petrašiūnas A., Podėnas S., Rimšaitė J., Sæther O. A., Spungis V. (2006). Checklist of Lithuanian Diptera. New and Rare for Lithuania Insect Species.

[B11733522] Pleskot Krzysztof, Tóth Mónika, Apolinarska Karina (2019). Distribution of subfossil chironomids (Diptera, Chironomidae) along a water depth gradient in the shallow Lake Spore, northern Poland. Journal of Limnology.

[B11743130] Pliūraitė V., Volskis R., Kangur M. (1999). Hydrobiological Research in the Baltic Countries. Part I. Rivers and Lakes.

[B11743088] Pliūraitė V. (2001). The seasonal change of macrozoobenthos in the Merkys river in 1998. Acta Zoologica Lituanica.

[B11743171] Pliūraitė V. (2001). Makrozoobentoso gausumo, biomasės ir rūšinės sudėties sezoninė kaita Merkio ir Šventosios upėse. Ekologija.

[B11743071] Pliūraitė V., Kesminas V. (2004). Species composition of macroinvertebrates in medium–sized Lithuanian rivers. Acta Zoologica Lituanica.

[B11733531] Płóciennik M. (2009). Three species of Chironomidae (Diptera) new for the Polish fauna. Lauterbornia.

[B11733540] Płóciennik M., Antczak O., Boulaaba S., Skonieczka M. (2015). First record of *Chironomuslongistylus* (Diptera, Chironomidae) from Poland. Lauterbornia.

[B11733549] Płóciennik Mateusz, Skonieczka Martyna, Antczak Olga, Siciński Jacek (2018). Phenology of non-biting midges (Diptera
Chironomidae) in peatland ponds, Central Poland. Entomologica Fennica.

[B11733558] Rocha L. G., Medeiros E. S.F., Andrade H. T.A. (2012). Influence of flow variability on macroinvertebrate assemblages in an intermittent stream of semi-arid Brazil. Journal of Arid Environments.

[B11733567] Ruginis T. (2007). Environmental impacts on macroinvertebrate community in the Babrungas River, Lithuania. EKOLOGIJA.

[B11743062] Sæther O. A., Spies M. (2005). Fauna Europea: Chironomidae. In de Jong H (ed.) 2005 Fauna Europea: Diptera Nematocera. Fauna Europea version 1.2. http://www.faunaeur.org.

[B11733576] Schartau A. K., Dolmen D., Hesthagen T., Mjelde M., Walseng B., Ødegaard F., Økland J., Økland K. A., Bongard T., Kålås J. A., Henriksen S., Skjelseth S., Viken Å. (2010). Environmental conditions and impacts for Red List species.

[B11733603] Serra Sónia R. Q., Cobo Fernando, Graça Manuel A. S., Dolédec Sylvain, Feio Maria João (2016). Synthesising the trait information of European Chironomidae (Insecta: Diptera): Towards a new database. Ecological Indicators.

[B11733594] Serra Sónia R. Q., Graça Manuel A. S., Dolédec Sylvain, Feio Maria João (2017). Chironomidae of the Holarctic region: a comparison of ecological and functional traits between North America and Europe. Hydrobiologia.

[B11733691] Centre SLU Swedish Species Information (2024). Swedish Species Observation System. https://www.artdatabanken.se/.

[B11733683] Spuņģis V., Kalniņš M. http://leb.daba.lv/Nematocera.htm.

[B11733613] Stur Elisabeth, Ekrem Torbjørn (2020). The Chironomidae (Diptera) of Svalbard and Jan Mayen. Insects.

[B11743156] Virbickas J., Pliūraitė V. (2002). The species composition of macrozoobenthos in small Lithuanian rivers. Acta Zoologica Lituanica.

